# Supplementary material for: Light dependent courtship behavior in Drosophila simulans and D. melanogaster
Source: PeerJ. 2020 Jul 16;8:e9499. doi: 10.7717/peerj.9499 (PMC7369021; doi:10.7717/peerj.9499)
Supplement: Supplemental Information 3 — The species, male and female strain identity, and treatment are provided in columns 1–4, respectively. Mean courtship latency is presented with standard deviation in parentheses. The corrected p-value for a Wilcoxon rank-sum test is also provided. Mean courtship effort and corrected p-values are presented similarly. The number of pairs with courtship latency/effort values recorded (N) is provided. [file peerj-08-9499-s003.docx]

| **Species** | **Male** | **Female** | **Treat-ment** | **Courtship Latency in minutes (Stdev)** | **corrected p-value** | **Courtship effort (Stdev)** | **corrected p-value** | **N** |
| --- | --- | --- | --- | --- | --- | --- | --- | --- |
| *D. melanogaster* | M-NG120 | M-NG120 | Light | 10 (4.16) | 0.14302 | 0.30 (0.23) | 0.5069 | 6 |
| *D. melanogaster* | M-NG120 | M-NG120 | Dark | 4.8 (5.72) |  | 0.20 (0.17) |  | 5 |
| *D. melanogaster* | M-BRAZ15 | M-BRAZ15 | Light | 0.9 (0.74) | 0.14302 | 0.64 (0.30) | 0.0578 | 10 |
| *D. melanogaster* | M-BRAZ15 | M-BRAZ15 | Dark | 4.88 (4.52) |  | 0.35 (0.25) |  | 8 |
| *D. simulans* | MAL261 | MAL261 | Light | 5.75 (7.54) | 0.5334 | 0.62 (0.15) | 0.0635 | 4 |
| *D. simulans* | MAL261 | MAL261 | Dark | 9.00 (4.18) |  | 0.28 (0.14) |  | 5 |
| *D. simulans* | SA169 | SA169 | Light | 9.25 (7.50) | 0.5334 | 0.68 (0.24) | 0.1333 | 4 |
| *D. simulans* | SA169 | SA169 | Dark | 20.5 (6.36) |  | 0.13 (0.14) |  | 2 |
| *D. simulans* | SA169 | MAL261 | Light | 5.00 (5.66) | 1 | 0.23 (0.11) | 0.7 | 3 |
| *D. simulans* | SA169 | MAL261 | Dark | 6.67 (6.43) |  | 0.50 (0.20) |  | 3 |
| *D. simulans* | MAL261 | SA169 | Light | 2.33 (2.31) | 1 | 0.70 (0.36) | 0.4724 | 2 |
| *D. simulans* | MAL261 | SA169 | Dark | 7.33 (7.51) |  | 0.43 (0.50) |  | 3 |

**Table S3. Courtship latency and courtship effort of D. melanogaster and D. simulans males when observed in the dark and when observed in the light.** The species, male and female strain identity, and treatment are provided in columns 1-4, respectively. Mean courtship latency is presented with standard deviation in parentheses. The corrected p-value for a Wilcoxon rank-sum test is also provided. Mean courtship effort and corrected p-values are presented similarly. The number of pairs with courtship latency/effort values recorded (N) is provided.
