# Supplementary figures and images for: Light dependent courtship behavior in Drosophila simulans and D. melanogaster
Source: PeerJ. 2020 Jul 16;8:e9499. doi: 10.7717/peerj.9499 (PMC7369021; doi:10.7717/peerj.9499)

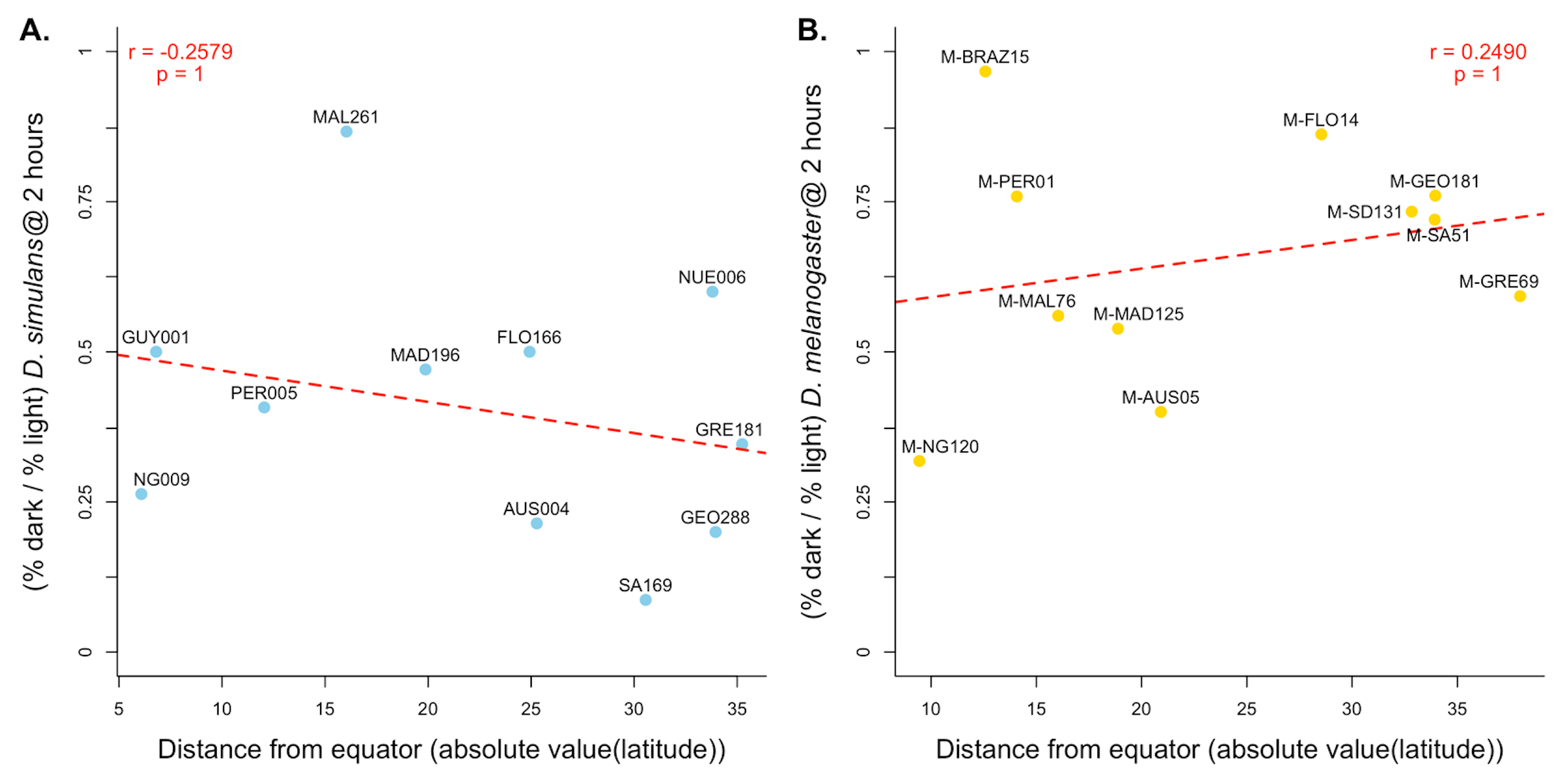

Supplement: Supplemental Information 4 — There is no correlation between relative light dependence at 2 hours (y-axis) and distance from the equator (absolute value of longitude, x-axis) for D. melanogaster strains (A.) or D. simulans strains (B.). For both, individual points are labelled with their strain label (Table 1). The red dashed line represents the best fit line from a linear model Pearson’s correlation coefficient and significance values, corrected for multiple comparisons, are displayed in the upper left corner of the plot. [file peerj-08-9499-s004.png]

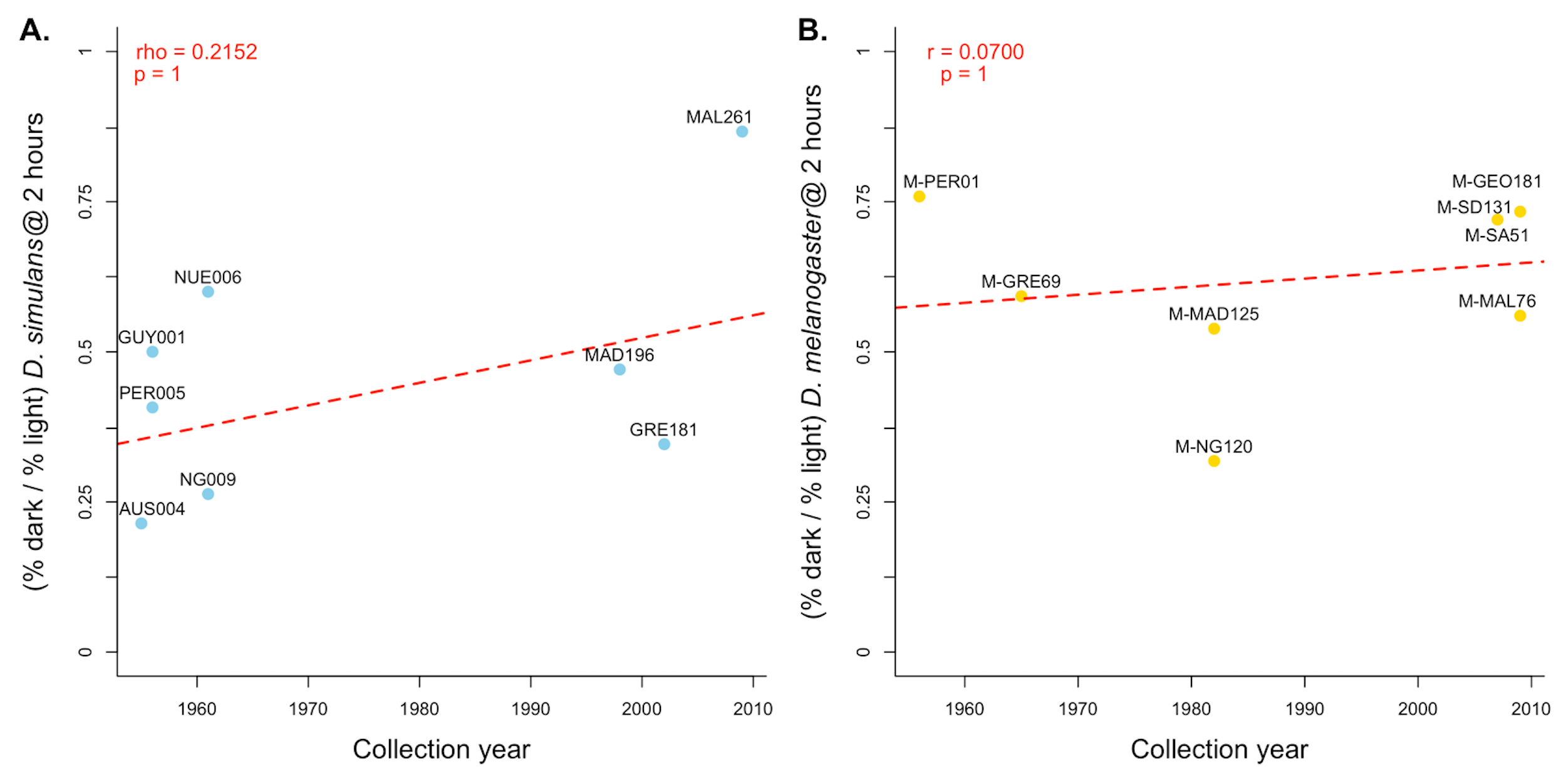

Supplement: Supplemental Information 5 — There is no correlation between relative light dependence at 2 h (y-axis) and collection year (x-axis) for D. melanogaster strains (A.) or D. simulans strains (B.). For both, individual points are labelled with their strain label (Table 1). The red dashed line represents the best fit line from a linear model Pearson’s correlation coefficient and significance values, corrected for multiple comparisons, are displayed in the upper left corner of the plot. [file peerj-08-9499-s005.png]
